# Effect of Sandblasting Pressure Combined with Acid Pickling on the Microstructure and Surface Properties of Ti-6Al-4V Alloy

**DOI:** 10.3390/ma19112256

**Published:** 2026-05-26

**Authors:** Yuanyuan Xie, Lei Li

**Affiliations:** School of Science, Inner Mongolia University of Technology, Aimin Sub-District, Xincheng District, Hohhot 010051, China; xieyy26411@163.com

**Keywords:** Ti-6Al-4V alloy, SLA treatment, surface modification, surface texturing, highly hydrophilic

## Abstract

Titanium alloys are widely used in aerospace, marine engineering, and biomedical fields owing to their excellent specific strength, corrosion resistance, and biocompatibility. As an important surface modification technique, sandblasting combined with acid pickling can not only eliminate the machining defects in Ti-6Al-4V but also improve its surface morphology, thereby playing a crucial role in enhancing its service performance. By employing advanced characterization equipment, including scanning electron microscopy (SEM), energy-dispersive X-ray spectroscopy (EDS), a 3D profilometer, and a friction and wear tester, combined with wetting theoretical models and morphological evolution analysis, this study systematically investigated the comprehensive effects of different pressure sandblasting followed by acid pickling on the surface microstructure, wetting behavior, and tribological properties of forged Ti-6Al-4V alloy. The results indicated that the combined application of sandblasting and acid pickling exerted a significant regulatory effect on the surface and interface characteristics of the Ti-6Al-4V alloy. After the combined sandblasting and acid pickling treatment, a distinct micro-pit network structure was formed on the surface of the Ti-6Al-4V alloy, and its hydrophilicity and roughness showed a positive correlation with the increase in sandblasting pressure. Notably, the microstructural evolution exhibited a high degree of internal consistency with the macroscopic wear resistance: the hierarchical micro-pit network exposed after acid pickling exerted an excellent “debris capture” effect, thereby suppressing the severe third-body abrasive wear observed in the solely sandblasted state. This study aims to enhance the surface roughness, wear resistance, and hydrophilicity of the Ti-6Al-4V alloy, providing a cost-effective and industrially applicable method for the surface texturing of titanium alloys.

## 1. Introduction

Titanium alloys play a crucial role in various fields, including aerospace, marine engineering, and biomedical implants, due to their exceptionally high specific strength, excellent corrosion resistance, and favorable biocompatibility [1,2,3,4,5]. However, they have inherent drawbacks, including relatively low hardness, poor resistance to shear deformation, and high susceptibility to adhesive wear [6,7,8,9]. Such inferior tribological performance severely limits their service life in complex relative sliding components. Furthermore, in specific engineering and biomedical application scenarios, surface wetting properties (e.g., hydrophilicity) also profoundly affect the material’s service performance [10]. Therefore, the use of a combined sandblasting and acid pickling process to synergistically regulate the tribological behavior and physicochemical properties of Ti-6Al-4V alloy has become a research focus in the current field of materials surface engineering [11].

Among various surface treatment technologies, sandblasting, as a low-cost physical modification process that is easy to industrialize and scale up, has been widely applied in the surface roughening and micro-texturing of titanium alloys. In recent years, numerous scholars have conducted extensive and in-depth research on the sandblasting process for titanium alloys, confirming its potential to enhance specific properties. Wennerberg A. et al. systematically compared the sandblasting effects of media with different particle sizes and found that the micro-scale rough morphology constructed under specific parameters could effectively increase the surface free energy, thereby significantly improving the initial wettability and liquid spreading ability of the titanium alloy interface [12]. Kim M. H. et al. used hard particles such as Al_2_O_3_ and SiO_2_ to perform high-energy impacts on the titanium matrix, successfully constructing a porous and rough structure with a large specific surface area. This mechanical interlocking effect significantly enhanced the bonding strength of subsequent coatings or biological bone tissues [13]. Furthermore, research by Sonntag R. et al. demonstrated that high-pressure sandblasting can induce severe plastic deformation in the surface layer of titanium alloys, introducing high-density dislocations and residual compressive stress, which, to a certain extent, improves the surface microhardness and resistance to fatigue crack initiation [14]. Gaikwad A. et al. attempted to use the micro-dimples generated by sandblasting to store lubricating media, aiming to improve the tribological behavior of titanium alloys [15].

However, with the in-depth development of research, the inherent limitations of the single sandblasting process have gradually been revealed. Pellegrini G. et al. objectively pointed out these limitations: high-energy impacts inevitably lead to the deep embedment or retention of hard abrasive particles (such as alumina) on the surface of the titanium alloy matrix. This not only may serve as a trigger point for local stress concentration but also may alter the local electrochemical potential, thereby inducing micro-galvanic corrosion [16]. Research by Baleani M. et al. directly confirmed that high-energy impacts not only induce surface microcracks in titanium alloys but also result in deeply embedded alumina fragments that directly act as “stress concentration zones.” Under cyclic loading or friction conditions, these areas are highly prone to becoming crack initiation sources, leading to premature material failure [17]. Similarly, Aparicio C. et al. confirmed from an electrochemical perspective that residual heterogeneous particles from sandblasting cause heterogeneity in the surface chemical composition of commercially pure titanium and explicitly pointed out that this heterogeneity changes the local potential distribution, thereby inducing micro-galvanic corrosion in body fluids or corrosive media and severely impairing the inherently excellent corrosion resistance of titanium alloys [18].

Given the aforementioned limitations induced by sole physical deformation, how to retain the beneficial macroscopic rough skeleton constructed by sandblasting while effectively eliminating heterogeneous abrasive contamination and passivating sharp microscopic stress concentration points has become an urgent engineering and scientific challenge. This inevitably requires the introduction of a highly selective post-treatment method; it is precisely against this background that acid pickling demonstrates its unique advantages.

Unlike purely physical mechanical impact, acid pickling mainly relies on the selective dissolution of the titanium matrix and its oxides by chemical solutions. As a crucial post-treatment step after sandblasting, it plays an irreplaceable role in surface reconstruction. In recent years, numerous scholars have conducted in-depth explorations on the application of the SLA (Sandblasted, Large-grit, Acid-etched) process on titanium alloy surfaces. Further fatigue mechanics analysis by Medvedev A. E. et al. revealed that although high-pressure sandblasting is highly likely to induce microcrack networks in the subsurface layer of titanium alloys, a moderate acid etching process can effectively remove this superficial brittle zone containing micro-defects [19]. Research by Orsini G. et al. confirmed that specific mixed-acid systems (e.g., HF/HNO_3_) can effectively remove residual heterogeneous hard particles from sandblasting and in situ reconstruct a more uniform and dense nascent TiO_2_ passivation film on the surface, thereby significantly enhancing the material’s corrosion resistance [20]. Le Guéhennec L. et al. found that the acid solution preferentially corrodes the titanium grain boundaries and the plastic deformation zones induced by sandblasting, successfully superimposing nanoscale sponge-like pores on the original rough skeleton; this micro-nano hierarchical structure greatly promotes the early proliferation and differentiation of osteoblasts [21]. Furthermore, through morphological evolution analysis, Adhitya K. et al. pointed out that the acid pickling process exerts a distinct “peak-clipping” and leveling effect on the sharp microscopic protrusions generated by sandblasting, which can passivate geometric stress concentration points to a certain extent [22].

However, although the traditional sandblasting combined with acid pickling (SLA) process has achieved remarkable success in surface purification and biocompatibility, most existing studies regard the SLA process as a “standard recipe” with fixed parameters for applications such as dental implants. Moreover, the performance evaluation of the traditional SLA process has focused heavily on cell adhesion and bioactivity, severely neglecting its tribological performance in complex relative sliding components. Faced with the inherently poor wear resistance of titanium alloys, the academic community has not yet clarified whether the surface morphology reshaped by SLA can suppress third-body abrasive wear under dry friction or boundary lubrication conditions, nor has there been effective exploration into whether the combined sandblasting and acid pickling process can effectively improve the surface wetting state.

In this study, forged Ti-6Al-4V alloy was used as the research object, and a treatment process of ‘different pressure sandblasting combined with acid pickling’ was proposed and systematically investigated to improve the surface roughness, wear resistance, and hydrophilicity of the Ti-6Al-4V alloy.

## 2. Materials and Methods

### 2.1. Materials Preparation

Forged Ti-6Al-4V alloy was selected for this experiment, and its chemical composition is shown in Table 1.

**Figure 1:**
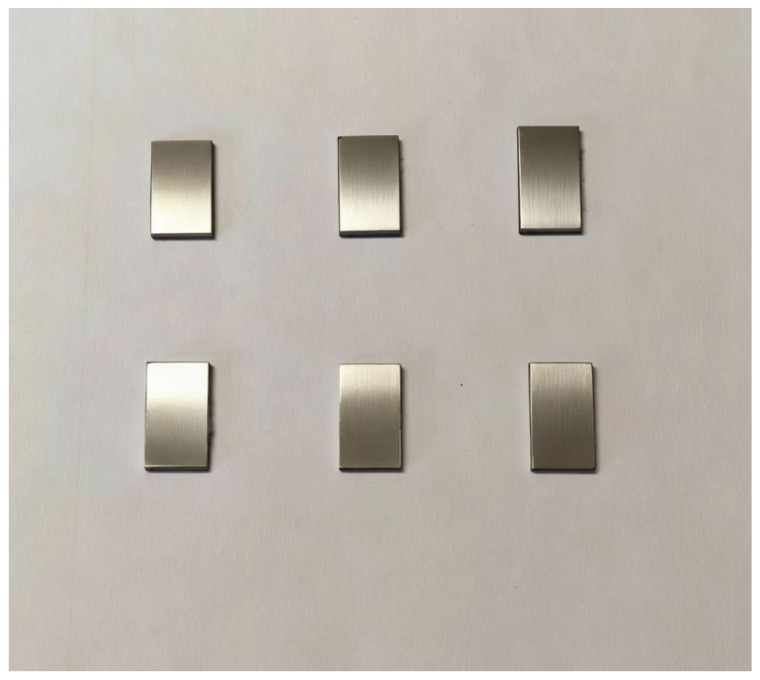
Physical image of Ti-6Al-4V specimen.

Ti-6Al-4V specimens (1.5 cm × 1 cm × 2 mm) were prepared from the alloy plate by wire cutting for subsequent processing. Figure 1 shows the physical appearance of the Ti-6Al-4V alloy.

### 2.2. Specimen Processing

To eliminate initial machining marks and ensure the consistency of the initial surface condition, all forged Ti-6Al-4V titanium alloy specimens were sequentially ground using 400#, 600#, 1000#, and 1500# silicon carbide (SiC) abrasive papers before surface modification. After grinding, the specimens were ultrasonically cleaned in deionized water to remove residual debris and oil stains and then dried for later use to ensure a smooth and clean substrate surface.

#### 2.2.1. Sandblasting

**Figure 2:**
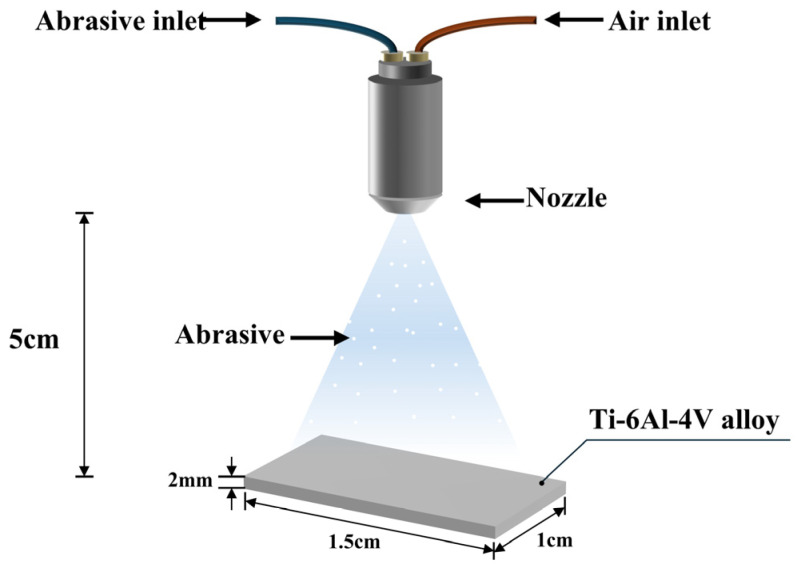
Schematic diagram of sandblasting.

**Figure 3:**
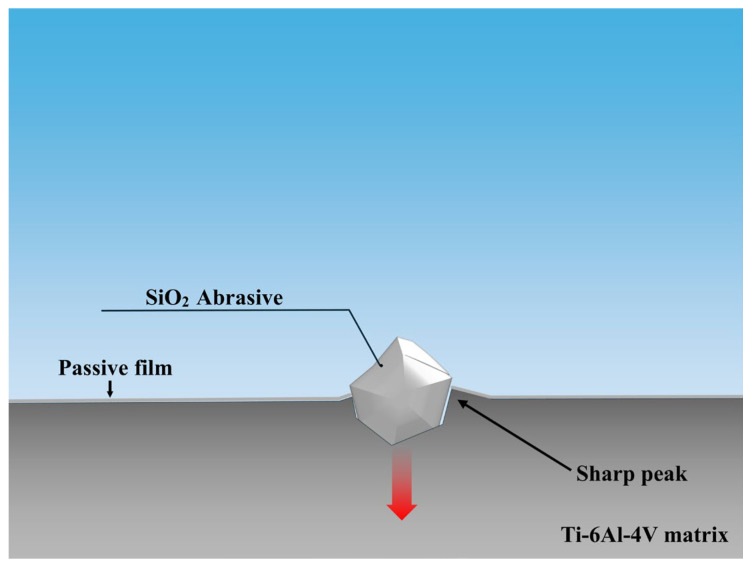
Principle diagram of sandblasting.

Figure 2 and Figure 3 present the schematic diagram and underlying working principles of the sand-blasting process. In this study, the pre-treated specimens were subjected to impact treatment using an AX-p3 dry sandblasting machine (Aixin Medical Equipment, Tianjin, China) at ambient temperature.

Quartz sand (SiO_2_) with a Mohs hardness of 7 and a grit size of 40–70# was selected as the hard impact abrasive; this abrasive exhibited an irregular, highly angular initial morphology prior to the blasting process. To ensure uniform surface plastic deformation, the sandblasting parameters were maintained constant as follows: a stand-off distance of 5 cm, an impact angle of 90°, a surface coverage of 100%, and a single treatment duration of 10 min. Three different sandblasting pressure groups were established: 0.2 MPa, 0.3 MPa, and 0.4 MPa, with the corresponding abrasive feed rates precisely controlled at 230 g/min, 345 g/min, and 460 g/min, respectively. Upon completion of the sandblasting process, the specimens were promptly retrieved, subjected to multiple rounds of ultrasonic cleaning, and thoroughly dried to maximize the removal of loose abrasive debris. Notably, driven by high-kinetic-energy impacts, the hard yet brittle SiO_2_ particles underwent severe fragmentation upon collision with the relatively ductile titanium matrix. Consequently, numerous fractured SiO_2_ micro-debris remained firmly and mechanically embedded within the plastically deformed surface layer, forming an abrasive-contaminated substrate that necessitated the subsequent chemical etching treatment.

#### 2.2.2. Acid Pickling

**Figure 4:**
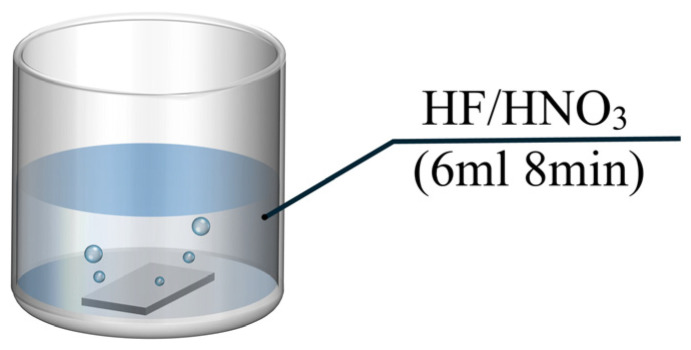
Schematic diagram of acid pickling.

**Figure 5:**
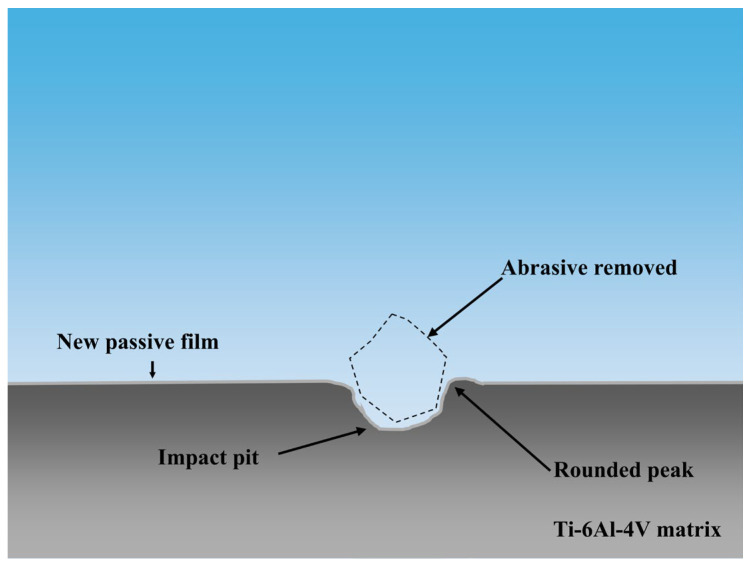
Principle diagram of acid pickling.

Finally, the sandblasted specimens were subjected to an acid pickling treatment at room temperature. The schematic and principle diagrams of pickling are shown in Figure 4 and Figure 5. A mixed acid solution composed of hydrofluoric acid (HF, 40 wt.%), nitric acid (HNO_3_, 65 wt.%), and deionized water at a volume ratio of 1:4:15 was prepared for the pickling process. To control the consistency and precision of the etching reaction, a fixed volume of 6 mL of the acid solution was allocated to each specimen group for 8 min of static pickling. After the reaction was completed, the specimens were quickly taken out and immediately immersed in deionized water for multiple rounds of ultrasonic cleaning. Finally, all processed specimens were dried in a drying oven. Based on the different sandblasting pressures and whether acid pickling was performed, the treated specimens were named original, Sb2, Sb3, Sb4, Sla2, Sla3, and Sla4, respectively. The specific groupings are detailed in Table 2.

### 2.3. Methods

#### 2.3.1. Surface Roughness and Topography

High-precision three-dimensional (3D) topography and surface roughness analyses of the specimens were carried out using an Olympus OLS4100 microscope (Olympus Corporation, Tokyo, Japan). The measurement was performed with a 20× objective lens, resulting in an evaluation area of 645 μm × 656 μm. A Gaussian filter was applied, and the roughness cut-off wavelength (*λc*) was set to 250 μm. The dedicated data analysis software (v3.1.10, Olympus LEXT OLS4100) equipped with the instrument was used to reconstruct the layer-by-layer images obtained during scanning into intuitive 3D surface topographies and to calculate the roughness parameters.

#### 2.3.2. Microstructure and Composition Characterization

The observation of the micro-morphology and the analysis of the surface chemical composition of the samples were mainly performed using a Thermo Fisher Apreo S LoVac scanning electron microscope (Thermo Fisher Scientific, Waltham, MA, USA). For the observation of surface morphology and fracture surfaces, the accelerating voltage was set to 15 kV, with a working distance of 4.86 mm and a beam current of 1.6 nA. The surface elemental composition was analyzed using energy-dispersive X-ray spectroscopy (EDS) mapping integrated with the SEM. Finally, post-processing software (v23.x, Thermo Scientific Pathfinder software) was used to analyze the acquired X-ray count pulses, generating EDS spectra and quantitative tables to characterize the surface micro-morphological features and the changes in surface elements caused by different treatment processes.

The micro-morphology of the wear tracks on the samples was observed using a Leica DM6 M microscope (Leica Microsystems, Wetzlar, Germany). The main purpose was to examine the surface wear morphologies under different processing parameters. Representative micrographs were obtained using the microscope’s proprietary image analysis software for subsequent analysis.

#### 2.3.3. Contact Angle Test

Water contact angle measurements were performed using a DSA25 contact angle goniometer (KRÜSS GmbH, Hamburg, Germany), at a temperature of 20 °C. The Ellipse (Tangent-1) fitting algorithm was adopted to accurately reflect the local spreading behavior of the droplets on the rough structures.

#### 2.3.4. Friction and Wear Test

The friction and wear performance of the samples was evaluated using an MFT-02-300N reciprocating friction and wear tester (Jkzc, Beijing, China). The tests were conducted in a ball-on-flat reciprocating sliding mode, using a GCr15 steel ball with a diameter of 6.35 mm as the counterpart. The test parameters included an 8 N normal load, 6 mm stroke, 5 Hz frequency, and 10 min duration. An integrated load sensor recorded real-time friction data to plot the coefficient of friction (*COF*) over time. After the test, the wear tracks were observed microscopically. To quantify the wear resistance, a geometric method was adopted. Assuming that the cross-section of the wear track is a circular segment due to the spherical counterpart, the average width ($\bar{W}$) of the middle section of the track was measured to calculate the wear volume ($\bar{V}$) using the following equation:

(1) V=A×S

where ***S*** is the reciprocating stroke. The cross-sectional area *A* is calculated from the wear scar width *W* and the sphere radius *R*. In this study, *R* corresponds to the geometric radius of the counterpart grinding ball, assuming that the cross-sectional profile of the wear track closely conforms to the rigid geometry of the sliding counterpart.

(2) $$A=R^{2}\arcsin\left(\frac{W}{2R}\right)-\frac{W}{2}\sqrt{R^{2}-\frac{W^{2}}{4}}$$

The wear rate *k* is then obtained from the following equation.

(3) $$k=\frac{V}{F\cdot L_{total}}$$

where *F* is the applied vertical load and *L_total_* is the total sliding distance. The tribological properties of the materials are comprehensively evaluated by comparing the friction coefficients and estimated wear rates of samples from different groups.

#### 2.3.5. Corrosion Resistance Test

The electrochemical corrosion behavior of the Ti6Al4V alloy specimens was evaluated using a CS350 electrochemical workstation (CorrTest, Wuhan, China) equipped with a standard three-electrode cell. The prepared specimens with an exposed area of 1.5 cm^2^ served as the working electrode (WE), while a platinum sheet and a saturated calomel electrode (SCE) were utilized as the counter electrode (CE) and reference electrode (RE), respectively. All electrochemical measurements were performed in a 3.5 wt.% NaCl solution at ambient temperature. Prior to testing, the specimens were immersed in the electrolyte for 1800 s to establish a stable open-circuit potential (OCP). Subsequently, electrochemical impedance spectroscopy (EIS) measurements were performed at the OCP with a sinusoidal amplitude of 10 mV over a frequency range of 100 kHz to 10 mHz. Finally, potentiodynamic polarization tests were conducted over a potential range of −0.5 V to +0.5 V (vs. OCP) at a constant scan rate of 1 mV/s.

## 3. Results

### 3.1. Surface Roughness Analysis

**Figure 6:**
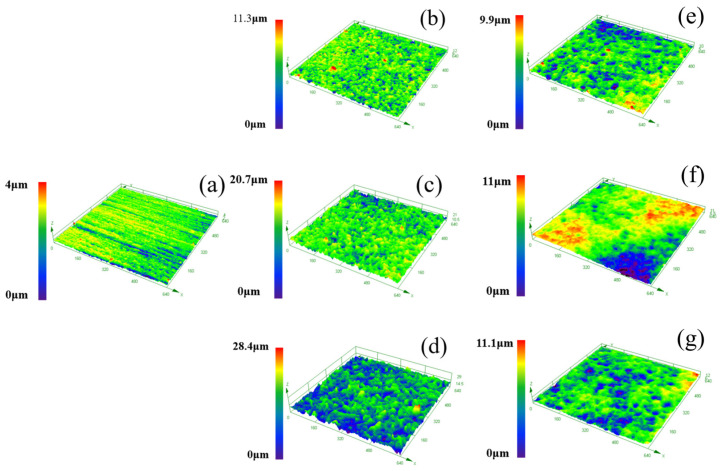
Morphologies before and after sandblasting at different pressures and subsequent acid pickling: (**a**) original state, (**b**) sandblasted at 0.2 MPa, (**c**) sandblasted at 0.3 MPa, (**d**) sandblasted at 0.4 MPa, (**e**) sandblasted at 0.2 MPa + acid pickling, (**f**) sandblasted at 0.3 MPa + acid pickling, (**g**) sandblasted at 0.4 MPa + acid pickling.

**Figure 7:**
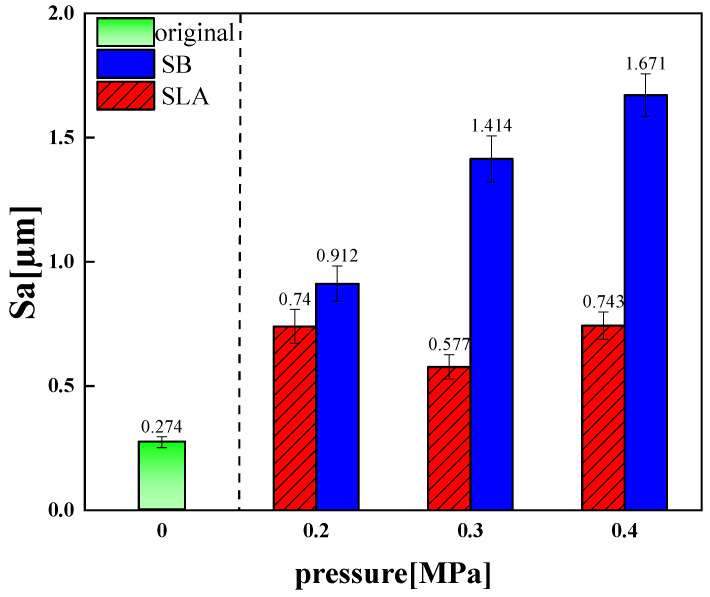
Roughness changes before and after sandblasting at different pressures and subsequent acid pickling.

Figure 6 and Figure 7 show the 3D surface morphologies and roughness changes in forged Ti-6Al-4V under different sandblasting pressures and subsequent acid pickling.

Sandblasting significantly increased the surface roughness compared with the pristine forged state. The *Ra* values showed a distinct pressure dependence, increasing monotonically from 0.274 µm to a maximum of 1.671 µm as the blasting pressure increased from 0.2 to 0.4 MPa. This increase is attributed to the higher kinetic energy of the abrasives, which caused intense plastic deformation, deeper impact craters, and enhanced peak-to-valley topographies [23,24].

After pickling, the *Ra* values of all sandblasted groups decreased, indicating a notable “smoothing effect.” The acid preferentially dissolved the high-energy micro-asperities and sharp edges induced by sandblasting, thereby reducing the arithmetical mean deviation of the profile [25]. Although *Ra* values quantitatively reflect macroscopic surface fluctuations, they fail to reveal microstructural details (e.g., microcracks, embedded abrasives, or etch pits) and surface chemical cleanliness. Therefore, subsequent micro-morphological (SEM) and chemical (EDS) analyses were necessary to clarify the physicochemical mechanisms driving this roughness evolution and to confirm the removal of sandblasting residues.

### 3.2. Micro-Morphology and Elemental Composition

The surface of the untreated Ti-6Al-4V alloy was flat with regular, directional parallel striations, which mainly originated from the machining marks left by precision cutting and mechanical polishing. This morphology, characterized by low micro-roughness and weak surface undulation, indicated limited surface plastic deformation and relatively low residual stress in the pristine state [26].

**Figure 8:**
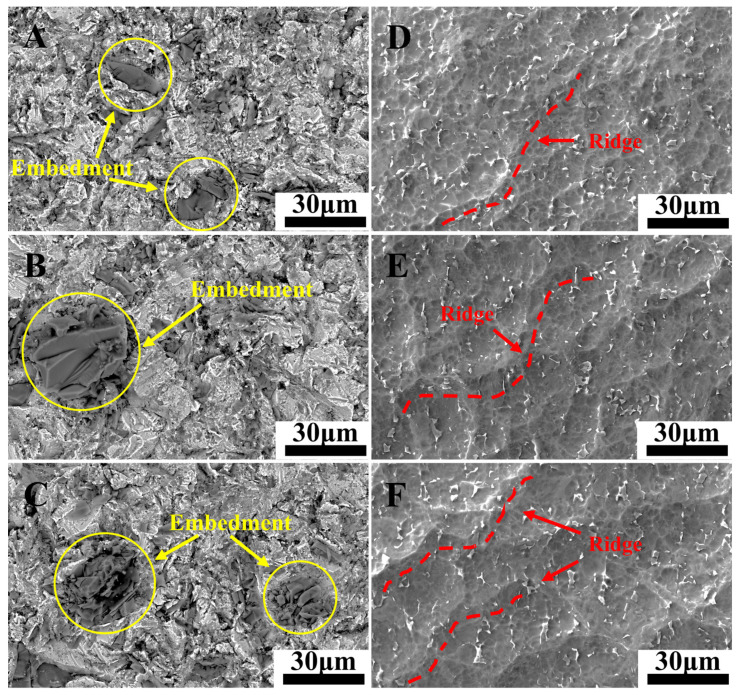
Surface morphologies before and after sandblasting at different pressures and subsequent acid pickling: (**A**) sandblasted at 0.2 MPa; (**B**) sandblasted at 0.3 MPa; (**C**) sandblasted at 0.4 MPa; (**D**) sandblasted at 0.2 MPa + acid pickling; (**E**) sandblasted at 0.3 MPa + acid pickling; (**F**) sandblasted at 0.4 MPa + acid pickling. Yellow markers indicate embedded SiO_2_, Red marks indicate ridge.

Figure 8 shows the surface micro-morphologies of the specimens. SEM analysis indicated that sandblasting at 0.2 MPa significantly changed the surface morphology of Ti-6Al-4V. The original parallel machining striations were mostly eliminated, replaced by irregular ridges and multi-directional grooves resulting from high-speed SiO_2_ particle impacts. This complex microtopography was driven by the repeated impact and cutting actions of the abrasives during sandblasting, which simultaneously increased the surface roughness and induced severe plastic deformation in the surface layer [27].

In addition, high-energy impacts caused some SiO_2_ abrasives to embed into the substrate surface. Increasing the blasting pressure to 0.3 MPa enhanced the kinetic energy of particles and intensified the impacts. This further strengthened the ridge and groove features, deepened localized plastic deformation, and increased both the quantity and size of the embedded particles [28].

Subsequent acid pickling further changed the surface morphology of the sandblasted specimens. This treatment completely eliminated mechanically embedded foreign abrasives and blunted the sharp protrusions induced by sandblasting, resulting in a more rounded profile. The acid preferentially dissolved the embedded SiO_2_, and the surrounding high-energy defect zones caused by severe plastic deformation. It also likely induced differential etching in different microstructural regions, further enhancing the microscopic surface undulations [29,30].

**Figure 9:**
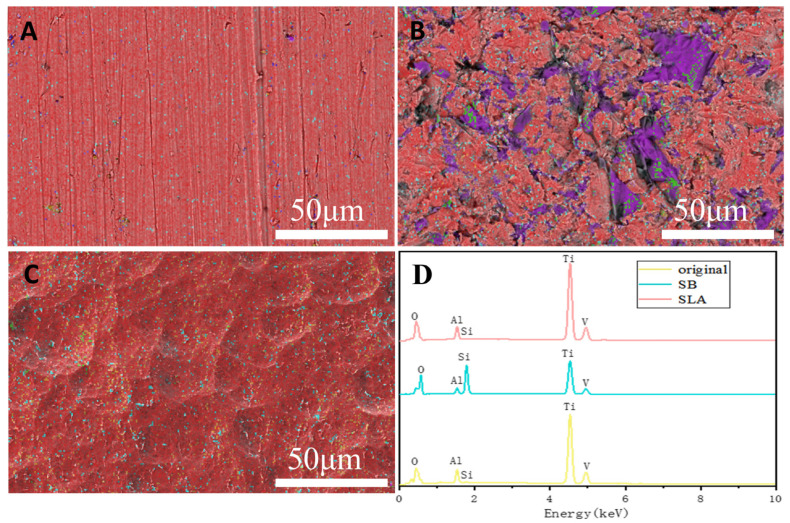
(**A**) Element mapping of original state; (**B**) Element mapping of specimen sandblasted at 0.4 MPa; (**C**) Element mapping of specimen sandblasted at 0.4 MPa + acid pickling; (**D**) EDS spectra of the three states under 0.4 MPa sandblasting. Red: Ti; Purple: Si; Cyan: V; Green: O; Brown: Al.

Figure 9 shows the EDS elemental mapping results. Sandblasting the pristine Ti-6Al-4V significantly increased the Si signal, indicating severe SiO_2_ embedment that was resistant to conventional cleaning. After acid pickling, the Si signal decreased significantly, confirming the effective removal of embedded SiO_2_. The detachment of these abrasives left distinct micro-pits at the impact sites, which, together with the retained sandblasting-induced ridges, formed a multi-scale rough structure. This morphology characterized the synergistic effect of sandblasting and acid pickling, highlighting the crucial role of acid pickling in eliminating foreign contaminants and exposing the truly deformed surface.

### 3.3. Surface Wettability Analysis

**Figure 10:**
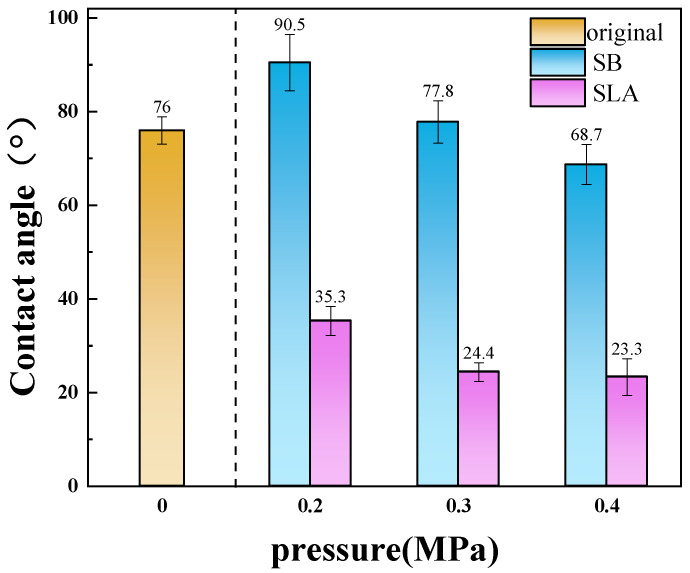
Surface static contact angle. Original: original state SB: sandblasted only; SLA: sandblasted + acid pickling.

The static contact angles of the Ti-6Al-4V alloy surfaces under different processing conditions are shown in Figure 10. The results revealed obvious synergistic effects, indicating that wettability is not only highly dependent on the type of treatment but also very sensitive to the sandblasting pressure. For the as-sandblasted samples, the surface wettability improved significantly as the blasting pressure increased. While the untreated sample had a contact angle of approximately 76°, the sample sandblasted at 0.2 MPa had the highest contact angle of about 90.5°, indicating near-hydrophobic behavior. In contrast, when the blasting pressure was increased to 0.4 MPa, the contact angle decreased significantly to 68.7°.

Although sandblasting at 0.2 MPa generated surface roughness, the formed impact craters were relatively shallow and isolated. These features easily trap air in the micro-pits to form an ‘air cushion’ consistent with the Cassie-Baxter model [31], thereby hindering droplet spreading. When the pressure was increased to 0.4 MPa, high-energy bombardment produced deeper and more densely packed hierarchical structures. Such extreme plastic deformation increased the effective surface area and simultaneously introduced a higher density of dislocations and lattice distortions [32], significantly enhancing the surface energy. Consequently, the wetting behavior shifted toward the Wenzel model, in which the increased roughness inversely amplified the inherent hydrophilicity of the substrate, resulting in a reduced contact angle [33].

After acid pickling treatment, the contact angles of all samples decreased sharply. This change revealed the dominant role of the surface chemical state in determining wettability: after pickling, the contact angle of the 0.4 MPa group decreased to a minimum of approximately 23.3°, while the 0.2 MPa group maintained a relatively higher angle of 35.3°.

Consistent with the EDS analysis, acid pickling completely removed the relatively hydrophobic embedded SiO_2_ and the initial contamination layer. The newly exposed clean titanium substrate easily forms a hydroxylated surface in aqueous environments [34]. This high-energy state increases the affinity for water molecules, enhances hydrophilicity, and provides an optimal interfacial environment for subsequent adhesive bonding, spray coating, and cell adhesion and proliferation.

Although acid pickling slightly reduced the macroscopic surface roughness, its peak-truncating effect effectively connected the isolated craters induced by sandblasting. Consequently, the deep topographical skeleton left by the 0.4 MPa pretreatment, superimposed with the dense array of nanoscale pores generated during the subsequent low-temperature acid pickling, formed an optimal dual-scale hierarchical rough structure. According to the Wenzel wetting model, this hierarchical topography significantly increased the specific surface area, thereby enhancing the intrinsic wettability of the Ti-6Al-4V alloy. Furthermore, the newly formed dense nanoscale pores acted as an extensive network of micro-capillaries. When a liquid droplet contacted this surface, strong capillary forces actively drew the fluid into these micro/nano cavities, accelerating the wetting and spreading of the droplets [35] and ultimately resulting in the highly hydrophilic state observed in the multi-treated specimens.

In conclusion, the hydrophilic performance of the Ti-6Al-4V surface is determined by the combination of a ‘pressure-induced micro-skeleton’ and ‘pickling-induced chemical activation.’ The high-pressure sandblasting (0.4 MPa) pretreatment constructed a topographical foundation with a high specific surface area. Subsequently, acid pickling served as an ‘activator’; by eradicating foreign abrasives and refining the pore structure, it promoted a remarkable transition from weak hydrophilic to high hydrophilic.

### 3.4. Tribological Behavior Analysis

**Figure 11:**
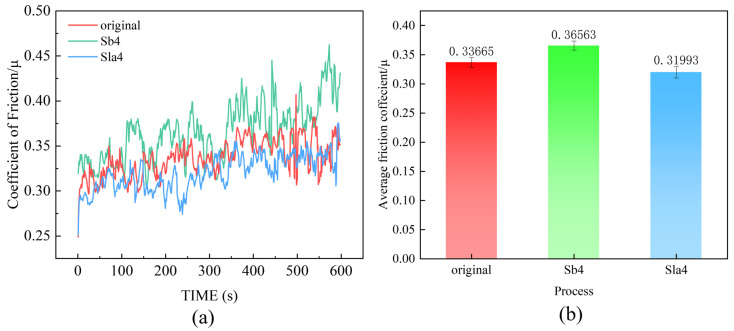
Influence of process on surface friction coefficient. (**a**) Friction coefficient curves, (**b**) Average friction coefficient.

**Figure 12:**
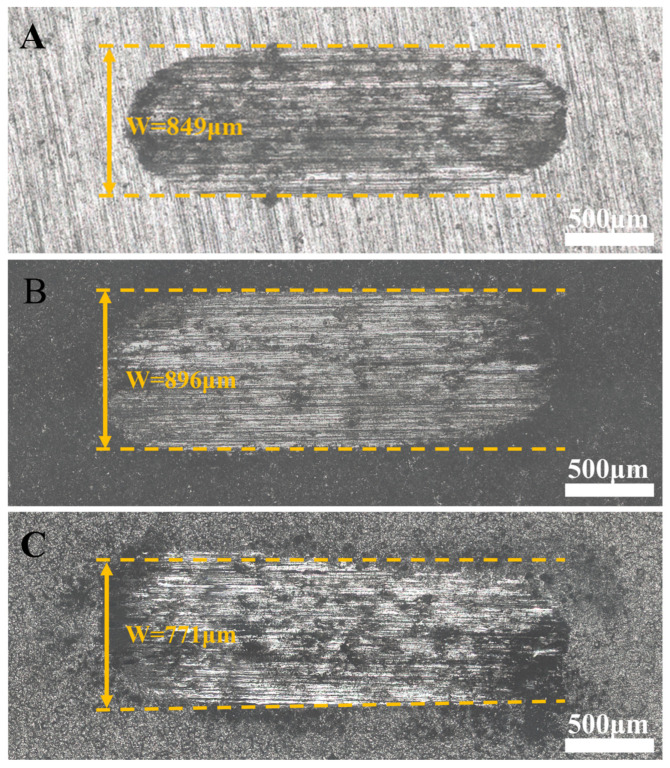
Friction and wear scar images. (**A**) original state; (**B**) sandblasted at 0.4 MPa; (**C**) sandblasted at 0.4 MPa + acid pickling.

The *COF* and wear tracks of the Ti-6Al-4V alloy under different treatment processes are shown in Figure 11 and Figure 12.

A comparison of the pristine, 0.4 MPa sandblasted, and 0.4 MPa sandblasted + pickled groups shows that sandblasting treatment significantly increased frictional resistance, while the subsequent acid pickling process minimized the coefficient of friction (*COF*) through morphological optimization. The data indicate that the as-sandblasted samples had the highest *COF*. This is mainly attributed to two factors: First, sandblasting generated numerous sharp ridge-like protrusions and high-frequency micro-irregularities on the surface. During the sliding of the friction pair, these sharp asperities severely collided and interlocked with the counterpart, significantly increasing plowing resistance and thus increasing the *COF* [36]. Second, as supported by EDS analysis, the embedded hard SiO_2_ abrasive debris may have detached during the sliding process, acting as third-body abrasives. This ‘abrasive wear’ mechanism further aggravated energy dissipation, resulting in significant fluctuations and the highest average value in the friction curve of the sandblasted state [37,38].

Notably, after acid pickling treatment, the coefficient of friction decreased to the lowest level among all groups, even lower than that of the pristine state. This friction-reduction effect originates from the ‘refining’ role of acid pickling on the surface morphology.

First, the acid effectively dissolved the sharp laps and brittle deformed layers generated by sandblasting, rounding the steep edges of the impact craters. This smooth microscopic transition significantly reduced the geometric resistance during sliding and decreased the collision frequency of the friction pair. Furthermore, the fully exposed impact pits after pickling played a crucial role during friction. These micro-pits act as ‘debris reservoirs,’ trapping the fine wear debris generated during sliding and thus preventing severe third-body wear at the contact interface [39,40,41].

Finally, the thorough removal of SiO_2_ particles eliminated a potential source of hard abrasives, returning the tribological system to pure metal-to-metal contact and stabilizing the friction environment. As shown in Figure 12, the micrographs of the wear tracks of the pristine, 0.4 MPa sandblasted, and 0.4 MPa sandblasted + pickled specimens show wear track widths of 849 μm, 896 μm, and 771 μm, respectively. This significant reduction in the wear track width of the complex-treated specimen further confirms the aforementioned analysis.

### 3.5. Corrosion Results Analysis

**Figure 13:**
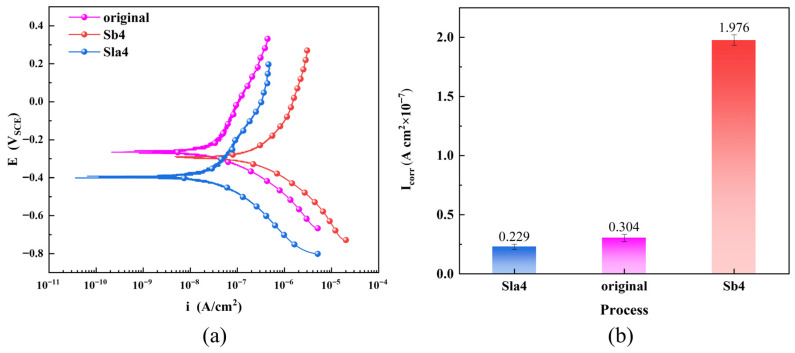
(**a**) PDP plots of Ti-6Al-4V subjected to different treatments in a 3.5 wt.% NaCl solution; (**b**) Average corrosion current densities of Ti-6Al-4V under various treatments.

Figure 13 presents the potentiodynamic polarization curves of the treated Ti-6Al-4V specimens, along with the average corrosion current densities under various conditions. All specimens exhibited nearly parallel cathodic branches, indicating that the primary reduction reactions occurring in the cathodic region shared identical kinetic mechanisms.

The purely sandblasted specimen (Sb4) exhibits pronounced active dissolution behavior, characterized by a significantly elevated anodic current density and the absence of a distinct, stable vertical passivation region. This accelerated and unimpeded dissolution is fundamentally attributed to the severe disruption of the native passive film, caused by the high density of stress-induced defects and embedded SiO_2_ impurities introduced during sandblasting. Specifically, the heterogeneous interfaces formed between the embedded SiO_2_ particles and the surrounding highly stressed titanium matrix trigger intense localized micro-galvanic corrosion [42]. Within these densely distributed micro-galvanic cells, the plastically deformed titanium matrix acts as the active anode, undergoing preferential and continuous dissolution.

However, following subsequent chemical etching, the Sla4 specimen demonstrates a markedly different trend. The anodic branch of Sla4 displays an exceptionally smooth and nearly vertical slope spanning a broad potential window. This curve trajectory signifies a drastic reduction in the anodic dissolution rate and a highly stable passive state. It concurrently demonstrates that the acid etching completely removed the embedded SiO_2_ particles and dissolved the highly stressed active layer, thereby eliminating the driving force for micro-galvanic corrosion [43]. The average corrosion current densities for the different treatments, derived via Tafel extrapolation, reveal that the corrosion current density of the Sb4 specimen is approximately an order of magnitude higher than that of the Sla4 specimen, further corroborating the aforementioned deductions.

**Figure 14:**
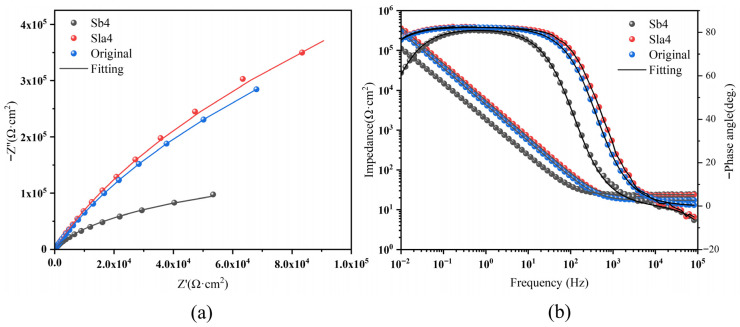
(**a**) Nyquist plots and (**b**) Bode plots for the specimens under various treatments in a 3.5 wt.% NaCl solution.

Figure 14 displays the Nyquist plots, as well as the Bode impedance and phase angle plots, for the treated Ti-6Al-4V specimens in a 3.5 wt.% NaCl solution. The experimental data exhibited a high degree of agreement with the simulated EIS curves. The radius of the capacitive arc corresponds to the magnitude of corrosion resistance. Visually, the capacitive arc radius of the Sla4 specimen was larger than that of the as-received state (Original), and both were significantly larger than that of the purely sandblasted state (Sb4). The impedance modulus (*|Z|*) at the lowest frequency (0.01 Hz) reflected the overall polarization resistance. The Sla4 specimen achieved the highest *|Z|* value, whereas the Sb4 specimen recorded the lowest. Furthermore, the peak phase angle of Sla4 was closest to −90°, confirming the superior capacitive response of its passive layer [44].

To interpret the acquired EIS spectra, a modified Randles equivalent circuit model, Rs(QRct), was employed for the fitting procedure. A constant phase element (CPE, denoted as Q) was used in place of a pure capacitor to compensate for the non-ideal dielectric behavior induced by the hierarchical micro-pits on the SLA-treated surfaces [45]. The Chi-square (*χ*^2^) values, representing the goodness-of-fit for all specimens, were strictly maintained in the order of 10^−4^.

The charge transfer resistance (Rct) characterized the barrier resistance of the oxide film against the penetration of aggressive ions. The Rct of the Sla4 specimen reached the highest value, demonstrating the robust corrosion resistance provided by the newly reconstructed passive film. Additionally, compared with the Sb4 specimen, the Y0 value of the Sla4 specimen exhibited a significant decrease. According to the parallel-plate capacitor model, this pronounced drop in capacitance strongly substantiated the formation of a denser passive film on the material surface, which effectively restricted the permeation of aggressive ions from the electrolyte [46]. The parameters obtained from the aforementioned fitting calculations are comprehensively summarized in Table 3.

In conclusion, based on the comprehensive analysis of the EIS and potentiodynamic polarization results, the corrosion resistance of the Ti-6Al-4V specimens followed a clear hierarchy: Sb4 < Original < Sla4. This thoroughly confirmed that the SLA composite process played a crucial role in removing embedded sandblasting particles and reconstructing a high-quality passive film.

## 4. Conclusions

This paper systematically investigates the synergistic regulation effects of sandblasting pretreatment under different pressures (0.2–0.4 MPa) and subsequent mixed acid pickling on the surface morphology, chemical composition, wettability, and tribological properties of as-forged Ti-6Al-4V titanium alloy. The main conclusions are drawn as follows:

Sandblasting forms irregular ridge-and-groove structures via severe plastic deformation, accompanied by mechanical embedding of micron-sized SiO_2_ abrasives. The subsequent acid pickling not only effectively and selectively removes the embedded heterogeneous SiO_2_ particles and the contaminated stress layer, but also transforms sharp micro-peaks and valleys into a multi-scale rough structure with smooth edges and good connectivity.

Surface roughness shows a significant positive correlation with sandblasting pressure. Although the “peak-cutting and valley-filling” effect of acid pickling leads to a substantial reduction in roughness for all specimens, the surfaces still inherit the macroscopic morphology gradient determined by sandblasting pressure.

Surface wettability undergoes a transition from “intrinsic state → hydrophobic tendency → high hydrophilicity”. The pure sandblasted surface traps air in sharp microstructures (consistent with the Cassie–Baxter model), resulting in an increased apparent contact angle (up to 90.5° at 0.2 MPa). After acid pickling, the wetting state shifts to the Wenzel model due to the exposure of the pure titanium matrix and enhanced capillary driving force from micro-pits, causing a drastic drop in contact angle. The 0.4 MPa acid-pickled group achieves excellent hydrophilicity with a contact angle of 23.3°.

Pure sandblasting induces the highest frictional resistance due to strong mechanical interlocking and third-body abrasive wear caused by SiO_2_. In contrast, the acid-pickled surface completely eliminates hard abrasives, and the dense micro-pit structure exerts an outstanding “debris reservoir” effect at the sliding interface, significantly reducing cutting resistance and yielding the lowest friction coefficient below that of the original state.

The acid pickling process effectively stripped away the surface defect layer introduced by sandblasting and eliminated the embedded abrasives, thereby blocking the micro-galvanic corrosion pathways between the heterogeneous particles and the titanium matrix. This resulted in a significant enhancement in corrosion resistance compared with the purely sandblasted state. Furthermore, the etching process facilitated the reconstruction of a dense passive film, enabling the charge transfer resistance of the Sla4 specimen to reach the highest value among the three treatment conditions.

In summary, high-pressure sandblasting (0.4 MPa) combined with acid pickling optimally synergizes the advantages of “physical impact pore formation” and “acid pickling finishing”. This provides an efficient and highly engineering-promising method for the multifunctional modification of titanium alloy surfaces to achieve high hydrophilicity, low friction and better corrosion resistance.

While this study provides comprehensive insights into the dual cleaning-morphology effect on the modified surfaces, further research is warranted to deepen the understanding in this field. Future investigations will focus on adapting these surface modification mechanisms to additively manufactured (SLM) Ti6Al4V alloys. Additionally, systematically exploring the influence of varying processing parameters—such as different sandblasting impact angles—on the comprehensive tribological performance, along with the evaluation of surface residual stress and coating adhesion strength, will be the subject of our subsequent studies.

## Figures and Tables

**Table 1:** Chemical composition of the Ti6Al4V (wt.%).

Ti	Al	V	Fe	O	C	N	H
bal	6.18	4.3	0.19	0.1	0.01	0.012	0.002

**Table 2:** Sandblasting and acid pickling process.

Sandblasting Pressure	Acid Pickling	Lab Number
0	No	original1
0	No	original2
0	No	original3
0.2 Mpa	No	Sb21
0.2 Mpa	No	Sb22
0.2 Mpa	No	Sb23
0.2 Mpa	Yes	Sla21
0.2 Mpa	Yes	Sla22
0.2 Mpa	Yes	Sla23
0.3 Mpa	No	Sb31
0.3 Mpa	No	Sb32
0.3 Mpa	No	Sb33
0.3 Mpa	Yes	Sla31
0.3 Mpa	Yes	Sla32
0.3 Mpa	Yes	Sla33
0.4 Mpa	No	Sb41
0.4 Mpa	No	Sb42
0.4 Mpa	No	Sb43
0.4 Mpa	Yes	Sla41
0.4 Mpa	Yes	Sla42
0.4 Mpa	Yes	Sla43

**Table 3:** Electrochemical parameters extracted from the corrosion tests in 3.5 wt.% NaCl solution.

Specimen	E_corr_ V	I_corr_ A cm^2^ × 10^−7^	I_p_ A cm^2^ × 10^−8^	R_s_ Ω cm^2^	R_ct_ kΩ cm^2^	CPE_dl_	
						Y0 Ω^−1^cm^−2^ sn × 10^−6^	n_dl_
Sla4	−0.393	0.229	0.909	31.32	4748.12	22.02	0.9239
Sb4	−0.292	1.976	1.683	35.73	444.73	66.47	0.9103
original	−0.263	0.304	0.729	25.98	4141.81	28.32	0.9178

## Data Availability

The original contributions presented in this study are included in the article. Further inquiries can be directed to the corresponding author.
